# Practical Nutritional Strategies to Attenuate Physiological Stress in Adolescent Soccer Players: A Comparative Trial of CoQ10 and Taurine

**DOI:** 10.3390/nu18142229

**Published:** 2026-07-09

**Authors:** Yousra Alsinani, Majid Al-Busafi, Hossein Shirvani

**Affiliations:** 1 Curriculum and Instruction Department, College of Education, Sultan Qaboos University, Muscat 123, Oman; 2 Physical Education Department, College of Education, Sultan Qaboos University, Muscat 123, Oman; 3 Exercise Physiology Research Center, Life Style Institute, Baqiyatallah University of Medical Sciences, Tehran 1435916471, Iran

**Keywords:** coenzyme Q10, taurine, soccer, oxidative stress, muscle damage, immune function, CD4/CD8 ratio, adolescent athletes

## Abstract

**Background/Objectives:** Intensified training in adolescent soccer players increases oxidative stress, muscle damage, inflammation, and immune suppression, but direct comparisons of nutritional countermeasures are lacking. This randomised, double-blind, placebo-controlled trial compared the effects of 14-day coenzyme Q10 (CoQ10) versus taurine supplementation on haematological, oxidative, muscle damage, inflammatory, hormonal, and immune biomarkers in under-19 soccer players undergoing three repeated 90 min Soccer Match Simulation (SMS) sessions. **Methods:** Twenty-four male players (age 17.9 ± 0.7 years) received placebo (n = 8), CoQ10 (300 mg/day, n = 8), or taurine (4 g/day plus 4 g pre-session, n = 8). Blood was collected at baseline (T0), post-first session (T1), post-third session (T2), and 24 h post-third session (T3). Biomarkers included creatine kinase (CK), lactate dehydrogenase (LDH), malondialdehyde (MDA), total antioxidant capacity (TAC), interleukins (IL-6, IL-10, TNF-α), cortisol, testosterone, CD4/CD8 ratio, immunoglobulins (IgA, IgG), and plasma volume (Dill–Costill). Data were analysed by two-way repeated-measures ANOVA. **Results:** CoQ10 was superior in reducing MDA (T2: 0.83 ± 0.02 vs. 1.24 ± 0.03 μmol/L, *p* < 0.001), LDH (434 ± 9 vs. 684 ± 12 U/L, *p* < 0.001), and cortisol (20.2 ± 0.6 vs. 30.4 ± 0.8 μg/dL, *p* < 0.001), and preserved the testosterone:cortisol ratio (24.5 ± 1.1 vs. 13.6 ± 1.0 × 10^−3^, *p* < 0.001). CoQ10 was more effective than taurine in lowering IL-6 at T2 (3.5 ± 0.2 vs. 3.9 ± 0.2 pg/mL, *p* = 0.03), whereas taurine was more effective in increasing IL-10 (7.5 ± 0.2 vs. 5.7 ± 0.2 pg/mL, *p* = 0.005). Both supplements preserved CD4 counts (CoQ10: 790 ± 13, taurine: 795 ± 14 vs. placebo: 680 ± 15 cells/μL, *p* < 0.01) and the CD4/CD8 ratio, as well as IgA and IgG levels, with no between-supplement differences for immune outcomes (*p* > 0.05). No adverse events occurred. **Conclusions:** For adolescent soccer players undergoing intensified training, CoQ10 may be preferred when the goal is reducing oxidative stress, muscle damage, and catabolic load; taurine may be preferred for targeted anti-inflammatory support (IL-10 elevation). Either supplement effectively attenuated changes in circulating immune biomarkers. These preliminary findings provide evidence-based guidance for targeted sports nutrition, pending confirmation in larger trials.

## 1. Introduction

Soccer is the world’s most popular sport, characterised by intermittent high-intensity activities. During a 90 min match, elite players cover 10–13 km with frequent sprints and directional changes [1]. For under-19 (U19) players, intensified training camps and congested fixture schedules place substantial physiological and psychological demands on developing athletes [2,3]. While regular moderate exercise enhances antioxidant defences, repeated high-intensity bouts without adequate recovery can increase oxidative stress, provoke systemic inflammation, impair immune function, and raise the risk of overtraining and upper respiratory tract infections [4,5,6].

The Soccer Match Simulation (SMS) protocol [7,8] is a validated field-based model that closely replicates match demands, including multidirectional movements and integrated skills. Three repeated 90 min SMS sessions within five days represent an intensified training period that challenges the body’s recovery capacity.

The repeated high-intensity efforts and eccentric actions inherent in soccer match play can overwhelm endogenous antioxidant defences, resulting in oxidative damage to lipids, proteins, and DNA, as well as impaired muscle function [9,10]. This exercise-induced oxidative burden is exacerbated when training sessions are performed with insufficient recovery, as in the current intensified training schedule.

Oxidative stress arises from an imbalance between reactive oxygen species (ROS) and endogenous antioxidants. Coenzyme Q10 (ubiquinone) is an endogenous lipophilic antioxidant localised in the inner mitochondrial membrane, serving as an electron carrier in ATP synthesis and a membrane-stabilising antioxidant [11,12]. Recent meta-analyses confirm that CoQ10 (≥300 mg/day for ≥14 days) significantly reduces post-exercise MDA, CK, and LDH [13,14].

Taurine, a sulphur-containing non-essential amino acid, plays critical roles in osmotic regulation, calcium homeostasis, and modulation of inflammatory signalling [15,16]. Unlike CoQ10, taurine scavenges hypochlorous acid, stabilises membranes, and influences the NF-κB pathway to suppress pro-inflammatory cytokines while enhancing anti-inflammatory responses [17,18]. It reduces exercise-induced IL-6 and TNF-α and increases IL-10 [18,19]—a profile distinct from CoQ10.

The immune system of adolescent athletes is particularly vulnerable to intensified training, manifesting as transient lymphopenia, reduced T-cell proliferative capacity, and impaired secretory IgA [4,5]. The CD4/CD8 ratio serves as a key indicator of immune homeostasis [20,21]. Intensified training can reduce CD4+ counts and the CD4/CD8 ratio [22], and exhaustive effort in soccer players induces T-cell redistribution [23,24].

Despite growing evidence, critical gaps remain: (i) most studies are in adults, with limited data on U19 players; (ii) no direct comparison of CoQ10 and taurine on a comprehensive multi-system biomarker panel exists; (iii) integrated analyses linking immune modulation to oxidative and inflammatory outcomes are lacking; and (iv) plasma volume shifts are rarely corrected. The present study addresses these gaps by comparing CoQ10 (300 mg/day) vs. taurine (4 g/day with pre-session bolus) on oxidative stress, muscle damage, inflammation, hormones, immune subsets, and immunoglobulins in U19 soccer players undergoing repeated SMS sessions, with plasma volume correction. We hypothesised that both supplements would attenuate negative effects, with CoQ10 superior in reducing oxidative damage and muscle enzyme leakage, and taurine more effective in modulating inflammatory cytokines. We further hypothesised that both would preserve the CD4/CD8 ratio and immunoglobulin levels.

To account for exercise-induced haemoconcentration, which can artefactually elevate concentration-dependent biomarkers, plasma volume changes were calculated using the Dill–Costill formula [25] and all relevant biomarkers were corrected accordingly.

## 2. Materials and Methods

### 2.1. Study Design

**Figure 1:**
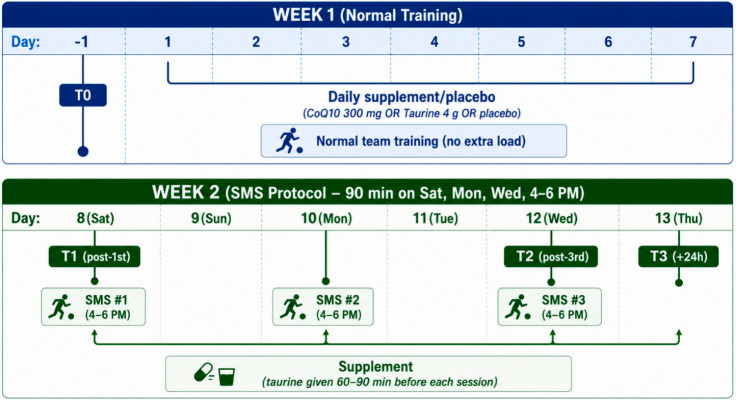
Schematic representation of the 14-day experimental protocol. T0: baseline blood draw (day −1). Days 1–7 (Week 1): daily supplementation (CoQ10 300 mg, taurine 4 g, or placebo) combined with normal team training. Days 8, 10 and 12 (Week 2): repeated 90 min Soccer Match Simulation sessions (SMS #1, #2, #3). Blood samples were collected immediately after SMS #1 (T1), immediately after SMS #3 (T2), and 24 h after SMS #3 (T3). Taurine group received an additional 4 g dose 90 min before each SMS session; CoQ10 and placebo groups continued their daily morning dose only.

A randomised, double-blind, parallel-group, placebo-controlled trial was conducted over 14 days. Participants were allocated via block randomisation (1:1:1) to three groups: placebo (n = 8), CoQ10 (n = 8), or taurine (n = 8). Figure 1 provides a schematic of the study timeline.

**Week 1 (days 1–7):** All participants consumed their assigned daily supplement (or placebo) and maintained their normal team training schedule (five sessions per week, approximately 60 min of moderate-to-vigorous intensity). Blood was drawn at baseline (T0) 24 h before the first supplement dose.

**Week 2 (days 8–14):** Supplementation continued. On day 8 (Saturday), day 10 (Monday), and day 12 (Wednesday), players performed a 90 min Soccer Match Simulation (SMS) protocol between 16:00 and 18:00. Blood was collected immediately after the first session (T1), immediately after the third session (T2), and 24 h after the third session (T3). No training occurred on days 9 (Sunday), 11 (Tuesday), and 13 (Thursday).

### 2.2. Participants

Thirty-two male under-19 soccer players from a single youth academy were assessed for eligibility. Inclusion criteria were: (i) age 16–19 years; (ii) training at least five times per week for the previous 12 months; (iii) no history of metabolic, cardiovascular, or immune disorders; (iv) no supplementation with antioxidants or amino acids for four weeks prior; (v) no current injury or illness; and (vi) non-smoker. Exclusion criteria were: any injury or medical condition that would preclude participation, use of medications affecting inflammation or hormonal status, and any prior adverse reaction to CoQ10 or taurine.

Of the 32 players assessed, eight were excluded before randomisation: five did not meet the inclusion criteria (two had taken antioxidant supplements within four weeks, two had a recent illness, one was outside the age range), two declined to participate, and one was excluded for other reasons (scheduling conflict). The remaining 24 players provided written informed consent from themselves and their legal guardians and were randomly assigned to three groups (n = 8 per group): placebo, CoQ10, or taurine.

Randomisation was performed by an independent researcher not involved in data collection or analysis, using a computer-generated random number sequence (www.randomizer.org). Allocation was concealed using sequentially numbered, opaque, sealed envelopes. Participants, coaches, outcome assessors, and the primary investigator were blinded to group allocation. The double-dummy technique ensured blinding was maintained throughout the study. The allocation code was revealed only after the final data analysis was completed.

Sample size was determined based on practical constraints of recruitment from a single youth academy and was not based on a formal a priori power calculation. Post hoc power analysis for the primary outcomes (MDA, LDH, IL-6) indicated observed power exceeding 0.85 for the main Group × Time interactions. However, given the modest sample size (n = 8 per group), the study was underpowered to detect small between-supplement differences for secondary immune parameters. As such, the findings should be interpreted as exploratory and hypothesis-generating, requiring replication in larger trials.

No participants withdrew or were lost to follow-up; all 24 completed the 14-day protocol and all blood sampling time points. Compliance with supplementation was high (mean 98.2%, range 94–100%). The study was approved by the institutional ethics committee (IR.BMSU.REC.1396.634) and conformed to the Declaration of Helsinki. A CONSORT flow diagram is presented in Supplementary Figure S1.

### 2.3. Maximal Aerobic Capacity Assessment: Queen’s College Step Test

Estimation of maximal oxygen consumption (VO_2_max) was conducted using the Queen’s College Step Test, a validated submaximal field test for predicting aerobic capacity in athletic populations [26]. The test procedure was as follows: participants stepped continuously onto and off a standard step box of 41.3 cm (16.25 inches) in height for three minutes at a cadence of 24 steps per minute (one complete step cycle—up, up, down, down—per second), regulated by a metronome. After completing the stepping protocol, the recovery heart rate was measured for 15 s (starting from 5 s post-exercise) and multiplied by four to obtain beats per minute [27]. Predicted VO_2_max (mL/kg/min) was calculated using the validated prediction equation for male participants:

VO_2_max (mL/kg/min) = 111.33 − (0.42 × heart rate (bpm))

All participants were tested in the morning (08:00–10:00) after a standardised light breakfast and at least 24 h after any vigorous exercise. The Queen’s College Step Test has been validated in soccer and volleyball populations, with reported predicted VO_2_max values for soccer players averaging 52.8 mL/kg/min, consistent with the values observed in the present cohort [26,27].

### 2.4. Supplementation Protocol

- **CoQ10 group:** 300 mg/day (three 100 mg capsules) of ubiquinone (Ubiquinol QU 10; Quest Vitamins (Quest Nutra Pharma), London, UK and Dubai, United Arab Emirates). This brand is a product of the Quest Nutra Pharma Group, which has manufacturing facilities in the United Kingdom and Dubai, operating to GMP standards. The supplement was supplied to the research team by Teb Andishan Pars Company (Tehran, Iran). The selected dosage of 300 mg/day is based on meta-analytic evidence showing that doses ≥ 300 mg/day for at least 14 days produce significant reductions in CK and LDH.
- **Taurine group:** 4 g/day (eight 500 mg capsules) of taurine (Eurovital Taurine 500 mg; Hakiman Tab Company, Tehran, Iran). This product is manufactured in Germany by Eurovital and distributed in Iran by Hakiman Tab Company. On training days (days 8, 10, 12), an additional 4 g dose was given 90 min before the SMS session; on non-training days, only the morning dose was taken.
- **Placebo group:** Identical-appearing capsules containing microcrystalline cellulose (supplied by Alborz Daroo Company, Tehran, Iran), administered on the same schedule as the active supplements.

To maintain blinding, all participants received the same number of capsules per dose using a double-dummy technique. Compliance was monitored by capsule counts and daily supplement logs returned each week. A participant was considered compliant if they consumed ≥90% of prescribed capsules.

### 2.5. Soccer Match Simulation (SMS) Protocol

The exercise protocol was based on the Loughborough Intermittent Shuttle Test described by Nicholas et al. [7] and subsequently modified by Russell, Benton, and Kingsley [8] to better replicate the multifaceted demands of competitive soccer. The modification incorporated backward and lateral movements, increased jogging volume, and a half-time break. Each session consisted of 90 min of intermittent activity divided into two 45 min halves separated by a 15 min half-time recovery period [8]. All movements were cued by a pre-recorded audio signal designed by the research team based on the published timing and sequence of the protocol [8]. The protocol included integrated assessments of soccer-specific skills, namely shooting, passing, and dribbling, as detailed in the original work [8].

**Figure 2:**
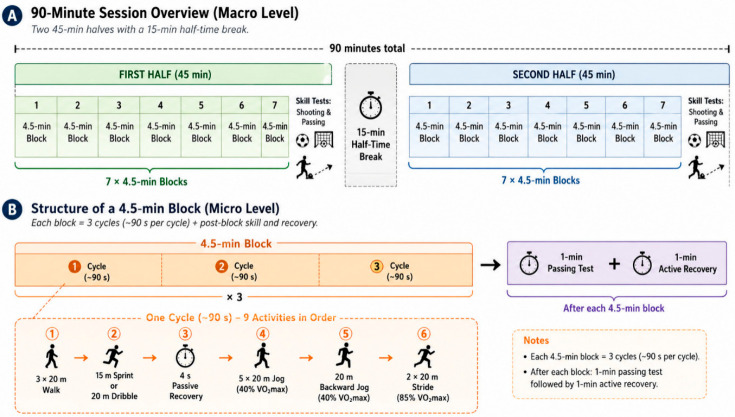
Schematic representation of the Soccer Match Simulation (SMS) protocol adopted from Russell et al. [8]. (**A**) A 90 min session comprises two 45 min halves separated by a 15 min half-time period. Each half consists of seven 4.5 min blocks of intermittent exercise. A 1 min shooting test and a 1 min passing test are performed at the end of each half. (**B**) Detailed structure of a single 4.5 min block. Each block contains three repeated cycles of the following sequence: three 20 m walks, an alternating 15 m sprint or 20 m dribble test, 4 s passive recovery, five 20 m jogs at 40% of individual VO_2_max, one 20 m backward jog at 40% VO_2_max, and two 20 m strides at 85% VO_2_max. After each block, a 1 min passing test is performed, followed by a 1 min recovery period. Movement speeds were individualised using each participant’s maximal multistage fitness test (MSFT) result.

The exercise was structured into repeating 4.5 min blocks, with seven such blocks completed per half [8]. Each block comprised three cycles of the following sequence: three 20 m walks, an alternating 15 m sprint or a 20 m dribble test, a 4 s passive recovery, five 20 m jogs at a speed equivalent to 40% of individual VO_2_max, one 20 m backward jog at 40% VO_2_max, and two 20 m strides at 85% VO_2_max. Movement speeds were calculated using each participant’s maximal multistage fitness test (MSFT) result. After each block, a 1 min passing test was performed, followed by a 1 min recovery period [8] (see Figure 2). Over the entire 90 min session, participants covered approximately 10.1 km and executed 56 passes, 16 shots, and 21 dribbles [8].

All sessions were conducted on a natural grass pitch of standard dimensions (105 m × 68 m) under consistent environmental conditions (ambient temperature 22–30 °C, relative humidity 28–35%). Before each session, participants completed a standardised 15 min warm-up consisting of light jogging, dynamic stretching, and soccer-specific movements. Heart rate was continuously monitored using Polar H10 chest strap monitors (Polar Electro, Kempele, Finland), and data were recorded and analysed with Polar Team Pro software (v2.6.0). Participants had ad libitum access to water during the recovery periods and at half-time.

### 2.6. Assessment of Habitual Dietary Intake

To control for the confounding effects of habitual diet on the measured physiological and immune biomarkers, a detailed assessment of macronutrient and caloric intake was performed [28,29]. Throughout the study period, participants completed a two-day food record (one training day and one rest day), with portion sizes estimated using a previously validated food frequency questionnaire adapted for athlete populations [30]. The collected data were analysed using the SAMAR nutritional analysis software (version 1.0; Behnampour et al., 2025), which has been validated for the Iranian food database [31]. A trained nutritionist reviewed each record with the participant to resolve any ambiguities. The analysis focused on total energy intake (kcal), absolute (g) and relative (g/kg body mass) intakes of carbohydrates, proteins and lipids, as well as saturated fatty acids (g) and cholesterol (mg). Mixed-model ANOVA was used to compare groups at baseline and post-intervention, as well as within each group across the study period.

### 2.7. Blood Sampling and Processing

Five millilitres of venous blood was drawn from the anterior forearm vein with the participant seated, after 10 min of quiet rest. All blood draws were performed between 16:00 and 18:00 to control for circadian variations. Samples were collected into three vacutainer tubes: (i) K_2_EDTA tube (3 mL) for complete blood count and flow cytometry; (ii) serum separator tube (2 mL) for hormone, immunoglobulin, and enzyme assays; and (iii) heparinised plasma tube (2 mL) for oxidative stress and cytokine assays. Blood samples were analysed within four hours for haematological parameters. For longer-term storage, plasma and serum were separated by centrifugation at 1500× *g* for 10 min at 4 °C, aliquoted into cryovials, and stored at −80 °C until batch analysis.

### 2.8. Biochemical and Immunological Assays

All assays were performed according to the manufacturers’ instructions, and samples from the same participant across all time points were analysed in the same batch to minimise inter-run variability. The details of each assay are summarised in Table 1.

### 2.9. Plasma Volume Correction

Individual percent changes in plasma volume (%ΔPV) from baseline were calculated using the Dill–Costill formula [25]:

%ΔPV = [ (Hgb_T0/Hgb_Tx) × ((1 − Hct_Tx)/(1 − Hct_T0))] − 1 × 100

where Hgb_T0 and Hct_T0 represent baseline haemoglobin and haematocrit values, and Hgb_Tx and Hct_Tx represent values at time point x (T1, T2, or T3). A negative percentage indicates plasma volume loss (haemoconcentration). All concentration-dependent biomarkers—including CK, LDH, cytokines (IL-6, IL-10, TNF-α), cortisol, testosterone, immunoglobulins (IgA, IgG), and CD4/CD8 cell counts—were multiplied by the correction factor (1 + %ΔPV/100) before statistical analysis.

### 2.10. Statistical Analysis

Data are presented as mean ± standard deviation (SD). Normality was assessed using the Shapiro–Wilk test, and homogeneity of variances was assessed using Levene’s test. Baseline demographic and physiological characteristics were compared between groups using one-way ANOVA.

For repeated measures, a two-way mixed ANOVA (Group × Time) was performed, with Time (T0, T1, T2, T3) as the within-subjects factor and Group (Placebo, CoQ10, Taurine) as the between-subjects factor. When sphericity was violated (Mauchly’s test), the Greenhouse–Geisser correction was applied. Significant main effects and interactions were explored using Tukey’s HSD post hoc test for pairwise comparisons. For between-group comparisons at individual time points, one-way ANOVA followed by Tukey’s HSD was used. For within-group comparisons across time points, repeated-measures ANOVA with Bonferroni correction was used.

The neutrophil-to-lymphocyte ratio (NLR) was calculated as the absolute neutrophil count divided by the absolute lymphocyte count. The CD4/CD8 ratio was calculated as the absolute CD4 count divided by the absolute CD8 count. Effect sizes are reported as partial η^2^ (eta squared). Statistical significance was set at *p* < 0.05 (two-tailed). All analyses were conducted using SPSS version 24.0 (IBM Corp., Armonk, NY, USA).

### 2.11. Data Availability and Trial Registration

The datasets generated during this study are available from the corresponding author upon reasonable request. This trial was not pre-registered; however, the study protocol was approved by the institutional ethics committee (IR.BMSU.REC.1396.634) and adheres to CONSORT guidelines.

## 3. Results

### 3.1. Participant Characteristics and Compliance

Baseline demographic and physiological characteristics are presented in Table 2. No significant differences were observed between groups for age, height, weight, or predicted VO_2_max from the Queen’s College Step Test (*p* > 0.05 for all). The mean VO_2_max values (52.3–53.1 mL/kg/min) are consistent with those reported for moderately trained youth soccer players [26,27].

Compliance with the supplementation protocol was high across all groups, with a mean adherence rate of 98.2% (range 94–100%). No adverse events were reported, and no participant withdrew from the study.

### 3.2. Dietary Intake Analysis

Dietary intake data are presented in Table 3. No significant differences were observed between the three groups at baseline or post-intervention, nor within each group across the study period (*p* > 0.05 for all comparisons). This confirms that habitual diet did not confound the observed biomarker responses, allowing the effects to be attributed to the supplementation intervention.

### 3.3. Plasma Volume Changes

Both supplements significantly attenuated exercise-induced haemoconcentration (Table 4). The placebo group showed progressive plasma volume loss across the three repeated 90 min SMS sessions, reaching a nadir of −15.1% at T2 (immediately after the third SMS session). CoQ10 produced the smallest plasma volume reduction at both T1 (−7.0%) and T2 (−8.5%), significantly different from both placebo (*p* < 0.001) and taurine (*p* = 0.02 for T1; *p* = 0.03 for T2). Taurine also significantly reduced plasma volume loss compared to placebo (*p* < 0.01 for both T1 and T2) but was less effective than CoQ10. By T3 (24 h after the third SMS session), plasma volume had returned toward baseline in all groups, with no significant differences.

All subsequent analyses of concentration-dependent biomarkers were performed on plasma volume-corrected values.

### 3.4. Oxidative Stress

MDA and TAC responses are summarised in Table 5. The placebo group exhibited a marked increase in MDA after the first SMS session (+90% at T1) with further elevation after the third session (+138% at T2). CoQ10 significantly blunted this rise, maintaining MDA at T2 at 0.83 ± 0.02 μmol/L—33% lower than the placebo group (*p* < 0.001) and 11% lower than the taurine group (*p* = 0.01). Taurine also reduced MDA compared to placebo (−25% at T2, *p* < 0.001) but to a lesser extent than CoQ10. TAC decreased progressively in the placebo group from baseline (1.26 ± 0.02 mmol/L) to T2 (0.95 ± 0.02 mmol/L, *p* < 0.001). CoQ10 preserved TAC throughout the protocol, with no significant decline from baseline at any time point (*p* > 0.05).

### 3.5. Muscle Damage Enzymes

Both CK and LDH increased substantially after repeated SMS sessions in the placebo group (Table 6). At T2, the placebo group exhibited CK levels of 514 ± 14 U/L (a 325% increase from baseline) and LDH levels of 684 ± 12 U/L (a 143% increase). CoQ10 supplementation resulted in significantly lower CK and LDH values at all post-exercise time points. At T2, CoQ10 reduced CK by 45% (284 ± 9 U/L) and LDH by 37% (434 ± 9 U/L) compared to placebo (both *p* < 0.001). Taurine also significantly reduced CK (−39%, 315 ± 10 U/L, *p* < 0.001) and LDH (−32%, 468 ± 11 U/L, *p* < 0.001) relative to placebo. Between-supplement comparisons revealed that CoQ10 was significantly more effective than taurine in lowering LDH at T2 (−7.3%, *p* = 0.03), whereas differences for CK did not reach statistical significance (*p* = 0.09).

### 3.6. Inflammatory Cytokines

Inflammatory cytokine responses are presented in Table 7. The placebo group showed the expected increase in pro-inflammatory IL-6 and TNF-α after exercise, with peak values at T2 (IL-6: 7.6 ± 0.3 pg/mL, +322%; TNF-α: 4.9 ± 0.2 pg/mL, +133%). Both supplements significantly attenuated these elevations. Notably, taurine was more effective than CoQ10 in lowering IL-6 at all post-exercise time points (at T2: 3.9 ± 0.2 vs. 3.5 ± 0.2 pg/mL, *p* = 0.03) and in elevating the anti-inflammatory cytokine IL-10. At T2, taurine increased IL-10 to 7.5 ± 0.2 pg/mL—a 14% increase compared to the placebo group (6.6 ± 0.3 pg/mL, *p* = 0.01) and a 32% increase compared to CoQ10 (5.7 ± 0.2 pg/mL, *p* = 0.005). For TNF-α, CoQ10 showed a modest but statistically significant advantage over taurine at T2 (3.3 ± 0.2 vs. 3.6 ± 0.2 pg/mL, *p* = 0.04).

### 3.7. Hormonal Responses

Table 8 presents the hormonal responses. In the placebo group, cortisol increased dramatically after repeated exercise, peaking at T2 (30.4 ± 0.8 μg/dL, a 117% increase from baseline), while testosterone declined (412 ± 12 ng/dL, a 22% decrease). Consequently, the testosterone-to-cortisol (T:C) ratio fell by 64% (from 37.5 ± 1.2 × 10^−3^ at baseline to 13.6 ± 1.0 × 10^−3^ at T2), indicating a profound catabolic state. CoQ10 significantly attenuated the cortisol rise (at T2: 20.2 ± 0.6 μg/dL, 34% lower than placebo, *p* < 0.001) and preserved testosterone levels (494 ± 9 ng/dL, 20% higher than placebo, *p* < 0.001). Consequently, the T:C ratio in the CoQ10 group at T2 was 24.5 ± 1.1 × 10^−3^—80% higher than the placebo group (*p* < 0.001) and 12% higher than the taurine group (*p* = 0.01). Taurine also improved the hormonal parameters relative to placebo but to a lesser degree than CoQ10.

### 3.8. Immune Markers (CD4, CD8, CD4/CD8 Ratio)

Table 9 presents the T-lymphocyte subset data. At baseline (T0), no significant differences were observed between groups for CD4 count, CD8 count, or the CD4/CD8 ratio (*p* > 0.05). Following the three repeated SMS sessions (T2), the placebo group exhibited a marked decline in CD4 count (from 851 ± 9 cells/μL at baseline to 680 ± 15 cells/μL, −20%, *p* < 0.001) and a reduction in the CD4/CD8 ratio (from 1.89 ± 0.07 to 1.94 ± 0.10, representing a 15% relative decline from baseline). In contrast, both supplement groups preserved CD4 counts at T2 (CoQ10: 790 ± 13 cells/μL; taurine: 795 ± 14 cells/μL), significantly higher than placebo (*p* < 0.001 for both), with no significant difference between the two active groups (*p* = 0.78). CD8 counts showed similar preservation (CoQ10: 416 ± 10 cells/μL; taurine: 412 ± 11 cells/μL; placebo: 350 ± 12 cells/μL; *p* < 0.01 for both comparisons). Consequently, the CD4/CD8 ratio remained stable in both supplement groups (CoQ10: 1.90 ± 0.09; taurine: 1.93 ± 0.09), whereas the placebo group showed a significant decline (*p* < 0.05 between placebo and both supplement groups). No significant differences were observed between CoQ10 and taurine for any immune parameter (*p* > 0.05).

### 3.9. Immunoglobulins (IgA and IgG)

Immunoglobulin responses are presented in Table 10. The placebo group showed a progressive decline in both IgA and IgG across the three SMS sessions, with IgA decreasing from 128 ± 4 mg/dL at baseline to 98 ± 5 mg/dL at T2 (−23%, *p* < 0.001) and IgG decreasing from 1155 ± 12 mg/dL to 925 ± 18 mg/dL (−20%, *p* < 0.001). Both CoQ10 and taurine significantly attenuated these declines. At T2, IgA levels in the CoQ10 (118 ± 4 mg/dL) and taurine (115 ± 5 mg/dL) groups were significantly higher than in the placebo group (both *p* < 0.001). Similarly, IgG levels in both supplement groups (CoQ10: 1080 ± 14 mg/dL; taurine: 1065 ± 15 mg/dL) were significantly higher than in the placebo group (both *p* < 0.001). No significant differences were observed between the two supplement groups for either immunoglobulin (*p* > 0.05).

### 3.10. Haematology and Leukocyte Differential

Table 11 presents the haematological parameters at T2 (peak cumulative stress). The placebo group exhibited marked haemoconcentration (elevated Hct and Hgb) and a pronounced shift in leukocyte differential, characterised by neutrophilia (78 ± 2%, a 34% increase from baseline), lymphopenia (13 ± 1%, a 50% decrease from baseline), and consequently an elevated neutrophil-to-lymphocyte ratio (NLR = 6.0 ± 0.6). CoQ10 significantly attenuated these changes, resulting in a normalised differential (neutrophils: 68 ± 2%, lymphocytes: 22 ± 1%) and a substantially lower NLR (3.1 ± 0.2, *p* < 0.001 vs. placebo). Taurine also improved the differential (neutrophils: 71 ± 2%, lymphocytes: 19 ± 1%, NLR: 3.9 ± 0.3) compared to placebo (*p* < 0.001) but was less effective than CoQ10 (*p* < 0.05 for all comparisons).

## 4. Discussion

This first direct comparison of CoQ10 and taurine in under-19 soccer players reveals distinct protective profiles. CoQ10 was superior in reducing oxidative stress, muscle damage (especially LDH), and cortisol-mediated catabolism, preserving the testosterone-to-cortisol ratio. Taurine was more effective in promoting an anti-inflammatory cytokine profile (↓ IL-6, ↑ IL-10). Both supplements preserved T-lymphocyte subsets and immunoglobulins, and reduced plasma volume loss.

### 4.1. Plasma Volume Correction: An Often-Overlooked Confounder

The Dill–Costill calculations revealed that the placebo group experienced a progressive plasma volume loss of up to 15% by T2. This degree of haemoconcentration would artificially elevate measured concentrations of muscle enzymes, hormones, and cytokines by approximately 15% if uncorrected, potentially leading to spurious conclusions about treatment efficacy [25]. Both supplements, particularly CoQ10, significantly reduced plasma volume loss, possibly through preservation of endothelial glycocalyx integrity or reduced capillary permeability. The fact that CoQ10 was more effective than taurine in this regard is novel and suggests a vascular protective effect beyond its antioxidant properties. These findings underscore the critical importance of plasma volume correction in exercise-based blood biomarker research, a practice that remains inconsistently adopted in the literature [32].

### 4.2. Oxidative Stress and Muscle Damage: CoQ10 as a Membrane Protector

The repeated 90 min SMS protocol induced substantial lipid peroxidation in the placebo group, with MDA increasing by 138% at T2. This level of oxidative stress is sufficient to impair muscle membrane integrity and delay recovery, as reflected by the 325% rise in CK and 143% rise in LDH. CoQ10 supplementation significantly attenuated these elevations, reducing MDA by 33% at T2 compared to placebo and by 11% compared to taurine. These findings are consistent with the recent 2025 meta-analysis by Qu and Qu [13], which demonstrated that CoQ10 significantly reduces MDA (MD = −0.61 μmol/L, *p* = 0.04), CK (MD = −71.81 IU/L, *p* = 0.012), and LDH (MD = −69.99 IU/L, *p* = 0.033) in athletic populations. Notably, the meta-analysis identified dose- and duration-specific effects, with significant reductions in LDH observed at 14 days of supplementation and in CK at doses ≥ 300 mg/day—precisely the parameters employed in the present study.

The mechanism underlying CoQ10′s superiority in attenuating muscle damage likely relates to its dual role as an electron carrier in the mitochondrial electron transport chain and as a direct antioxidant within the inner mitochondrial membrane [11]. By stabilising the mitochondrial membrane and reducing ROS generation during oxidative phosphorylation, CoQ10 may protect against the downstream effects of oxidative stress, including lipid peroxidation and sarcolemmal disruption. Taurine also reduced MDA, CK, and LDH to a lesser extent, consistent with its antioxidant properties (scavenging hypochlorous acid, upregulating antioxidant enzymes via the Nrf2 pathway) [16,18]. However, taurine’s effects on muscle damage markers were less pronounced, likely because its primary mechanisms—membrane stabilisation through osmotic regulation and modulation of calcium handling—complement rather than replace mitochondrial protection.

### 4.3. Inflammatory Modulation: Taurine as an Immune Signal Modulator

Perhaps the most striking finding of this study was the divergent effect of the two supplements on the inflammatory cytokine profile. Taurine uniquely elevated IL-10—the prototypical anti-inflammatory cytokine—by 14% compared to placebo and 32% compared to CoQ10 at T2, while simultaneously lowering IL-6 more effectively than CoQ10. This pattern is consistent with the known ability of taurine to suppress NF-κB activation and upregulate IL-10 production, as reviewed by Keshavarzi et al. [17], who noted that taurine influences pathways such as Nrf2/OH-1 and PI3-kinase/AKT while suppressing pro-inflammatory cytokines (TNF-α, IL-1β, IL-6). The biological significance of this shift is considerable: IL-10 not only suppresses pro-inflammatory cytokine synthesis but also promotes regulatory T-cell differentiation and supports tissue repair [33].

CoQ10, in contrast, had a modest anti-inflammatory effect (reducing IL-6 and TNF-α) but did not increase IL-10. This suggests that CoQ10′s primary anti-inflammatory action is indirect, arising from reduced oxidative damage and subsequent attenuation of damage-associated molecular pattern (DAMP) signalling that triggers inflammation, rather than direct modulation of immune signalling pathways [34]. For athletes engaged in back-to-back training sessions, the ability to enhance IL-10 may accelerate the resolution of inflammation and reduce the duration of post-exercise recovery windows, an effect that taurine appears uniquely positioned to provide.

### 4.4. Hormonal Responses: CoQ10 Preserves Anabolic Balance

The testosterone-to-cortisol ratio is a widely used, albeit imperfect, marker of anabolic/catabolic balance [35]. In the placebo group, the T:C ratio fell by 64% at T2, indicating a profound catabolic shift that, if sustained, could impair muscle repair, reduce training adaptation, and increase overtraining risk. CoQ10 supplementation attenuated the cortisol rise by 34% and preserved testosterone levels, resulting in a T:C ratio that was 80% higher than placebo at T2. To our knowledge, this is the first study to demonstrate that CoQ10 preserves the T:C ratio in adolescent soccer players under intensified training conditions.

Several mechanisms may explain this effect. Firstly, by reducing oxidative stress and muscle damage, CoQ10 may lower the inflammatory burden that stimulates cortisol secretion via the hypothalamic–pituitary–adrenal (HPA) axis [36]. Secondly, CoQ10’s role in mitochondrial ATP production may support the energy-intensive process of testosterone synthesis in the Leydig cells of the testes, which has a high demand for mitochondrial energy [37]. Thirdly, CoQ10 may reduce exercise-induced glucocorticoid receptor activation through its membrane-stabilising properties. Taurine also improved the T:C ratio relative to placebo but was less effective than CoQ10, aligning with the hypothesis that cortisol attenuation is primarily an outcome of reduced oxidative and inflammatory stress rather than direct HPA axis modulation.

### 4.5. Immune Function: Both Supplements Protect T-Lymphocyte Subsets

A key contribution of this study is the comprehensive assessment of T-lymphocyte subsets (CD4, CD8, CD4/CD8 ratio) and immunoglobulins in response to supplementation. The placebo group exhibited a 20% decline in CD4 count at T2, consistent with the well-characterised phenomenon of exercise-induced immunodepression [4,5]. In professional soccer players, long-term training has been shown to alter CD4+ and CD8+ cell concentrations, with a decrease in the CD4/CD8 ratio observed over the course of a competitive season [22]. Similarly, exhaustive effort in soccer players has been shown to induce CD4+ T-cell differentiation and increase T-regulatory cell percentages, reflecting immune redistribution rather than uniform suppression [23].

Both CoQ10 and taurine fully preserved CD4 counts, CD8 counts, and the CD4/CD8 ratio at T2, with no significant differences between the two active groups. This immune-protective effect likely arises from the combined reduction in oxidative stress and inflammation, both of which can suppress lymphocyte proliferation and function [38]. By lowering the post-exercise inflammatory burden, both supplements may prevent the lymphocyte apoptosis and redistribution that underlie exercise-induced lymphopenia [39]. The preservation of CD4 counts—critical for helper T-cell function and adaptive immunity—has clinical relevance for adolescent athletes, who face elevated risks of upper respiratory tract infections during periods of intensified training [6].

Immunoglobulin preservation followed a similar pattern. The placebo group showed a 23% decline in IgA and a 20% decline in IgG at T2, reflecting impaired humoral immunity. Both supplements attenuated these declines, with no differences between them. Given that secretory IgA is the first line of mucosal defence against respiratory pathogens, maintaining IgA levels during intensified training is a meaningful clinical outcome [40].

### 4.6. Haematological Parameters and NLR

The neutrophil-to-lymphocyte ratio (NLR) has emerged as a practical marker of systemic stress and recovery capacity in athletes [41]. The placebo group’s elevated NLR (6.0) at T2 reflects a stress response characterised by neutrophilia (from splenic demargination and cortisol-induced release) and lymphopenia (from lymphocyte redistribution and apoptosis). CoQ10 normalised the NLR to 3.1, while taurine reduced it to 3.9 (both *p* < 0.001 vs. placebo). The superior effect of CoQ10 on NLR is consistent with its greater ability to lower cortisol and preserve lymphocytes. The NLR may therefore serve as a simple, cost-effective marker for monitoring training load and recovery in field settings.

### 4.7. Comparison with Other Supplementation Strategies in Team Sports

The effect sizes observed in this trial compare favourably with other common ergogenic aids. For example, vitamin C/E combinations have shown inconsistent or even pro-oxidant effects in athletes, whereas CoQ10 produced consistent reductions in MDA and LDH. Omega-3 fatty acids primarily modulate inflammation (similar to taurine) but have less impact on oxidative stress markers [34]. Creatine monohydrate improves repeated-sprint performance but does not directly affect immune subsets or cortisol responses. Thus, CoQ10 and taurine offer unique, complementary benefits not fully captured by other supplements.

### 4.8. Dietary Control and Its Importance

An important strength of this study is the rigorous assessment of dietary intake using two-day food records analysed with the SAMAR nutritional analysis software (version 1.0; Behnampour et al., 2025), which has been validated for the Iranian food database [31]. The absence of significant differences in calorie, macronutrient, saturated fat, or cholesterol intake between groups or across the study period (Table 3) allows us to confidently attribute the observed biomarker changes to the supplementation rather than to dietary variation. This is particularly critical in adolescent athletes, whose dietary habits can be erratic and may confound exercise nutrition studies [28,29]. The mean energy intake (~2750 kcal) and carbohydrate intake (~4.9 g/kg) align with recommendations for youth soccer players during training periods [42].

### 4.9. Comparison with the Previous Literature

Our findings extend the existing literature in several important ways. Previous studies have examined CoQ10 primarily in adult athletes, with mixed results that may be partly attributable to inadequate dosing or duration. The present study confirms, in a youth population, that 300 mg/day for 14 days produces consistent reductions in MDA, CK, and LDH, aligning with the meta-analytic findings of Qu and Qu [13] and Talebi et al. [14]. For taurine, our results are consistent with recent narrative reviews demonstrating that taurine consistently reduces CK, LDH, and MDA after exercise, particularly 24–48 h post-effort, and may influence pain perception up to 96 h [19]; additionally, acute taurine supplementation has been shown to improve repeated-sprint performance in challenging environmental conditions [16].

The novel contributions of this study include: (i) the first direct comparison of CoQ10 and taurine in adolescent soccer players; (ii) the incorporation of a complete T-lymphocyte subset panel (CD4, CD8, CD4/CD8 ratio) alongside immunoglobulins; (iii) the application of plasma volume correction to all concentration-dependent biomarkers; (iv) the use of a validated, repeated 90 min Soccer Match Simulation (SMS) protocol [7,8]; (v) the integrated assessment of oxidative, inflammatory, hormonal, and immune outcomes under the same experimental conditions; and (vi) the rigorous control of dietary intake using a culturally appropriate nutritional analysis tool.

### 4.10. Limitations and Future Directions

Several limitations should be acknowledged. The primary limitation is the modest sample size (n = 8 per group). While post hoc power calculations for the primary outcomes (MDA, LDH, IL-6) exceeded 0.85, the study was underpowered to detect small between-supplement differences for secondary immune parameters (e.g., CD4/CD8 ratio). The high number of biomarker comparisons and the modest sample size increase the risk of type I error. While Bonferroni correction was applied within biomarker families, the exploratory nature of this study should be considered when interpreting the findings.

Additional limitations include: (i) the 14-day supplementation period does not address longer-term effects, and we did not verify supplement blood levels or compliance via pharmacokinetic measures; (ii) the absence of a combined CoQ10+taurine arm; (iii) no direct performance outcomes; (iv) predicted VO_2_max rather than direct gas analysis, and the use of a non-sport-specific test (Queen’s College Step Test) for exercise intensity prescription, rather than a sport-specific maximal test such as the Yo-Yo Intermittent Recovery Test; (v) single-centre, male-only sample limiting generalisability; (vi) peripheral blood CD4/CD8 ratio may not fully reflect functional immune status; (vii) the Dill–Costill formula has inherent assumptions; (viii) the differential acute dosing strategy for taurine confounds direct comparison with CoQ10; (ix) lack of formal maturation/puberty assessment (e.g., Tanner staging), which may influence hormonal and inflammatory responses in adolescent athletes; and (x) habitual training load was not quantified beyond the described schedule, and we did not assess sleep quality, psychological stress, or other lifestyle factors that may influence immune function.

Future research should conduct larger, multi-centre trials with longer supplementation, include a combination arm, incorporate functional immune outcomes (e.g., infection rates, vaccine responses), measure performance, and explore sex-specific responses.

## 5. Conclusions

Fourteen days of CoQ10 (300 mg/day) or taurine (4 g/day with pre-session bolus) in under-19 soccer players undergoing three repeated 90 min match simulations produced divergent but complementary protective profiles. CoQ10 was more effective in mitigating oxidative stress, muscle damage (particularly LDH), and cortisol-mediated catabolism, thereby preserving the testosterone-to-cortisol ratio. Taurine was more effective in promoting an anti-inflammatory cytokine profile (greater IL-10 elevation), while CoQ10 showed superior IL-6 reduction. Both supplements attenuated exercise-associated changes in selected circulating immune biomarkers (CD4, CD8, CD4/CD8 ratio, IgA, IgG), attenuated plasma volume loss, and normalised the neutrophil-to-lymphocyte ratio, with no significant differences between the two active groups for immune parameters.

For practitioners, the choice of supplement may be guided by the primary physiological challenge, although these recommendations should be considered preliminary: CoQ10 may be preferred during phases of high oxidative and muscle damage demands, while taurine may be preferred when rapid resolution of inflammation is critical. However, these recommendations are based on biomarker responses and require validation in larger trials with direct performance and recovery outcomes. A combined supplementation strategy warrants investigation to harness the complementary benefits of both agents. These findings support the potential usefulness of targeted, evidence-informed use of CoQ10 and taurine in adolescent soccer players, pending confirmation in larger, adequately powered studies.

## Figures and Tables

**Table 1:** Biomarker measurement methods and analytical specifications.

Domain	Marker	Method/Principle	Kit/Instrument	Assay Range	CV (%)
**Haematology**	RBC, WBC, Hct, Hgb, Plt, differential	Automated impedance/fluorescence	Sysmex XN-1000 (Sysmex, Kobe, Japan)	‒	<2
**Muscle damage**	CK	Enzymatic colorimetric (IFCC method)	Pars Azmoon Biochemistry kit (Pars Azmoon, Tehran, Iran)	10–2000 U/L	3.2
	LDH	Enzymatic colorimetric (IFCC method)	Pars Azmoon Biochemistry kit (Pars Azmoon, Tehran, Iran)	20–1500 U/L	2.9
**Oxidative stress**	MDA	TBARS assay (spectrophotometric)	Teb Pazhouhan Razi MDA kit (Teb Pazhouhan Razi, Tehran, Iran)	0–75 μmol/L	5.1
	TAC	ABTS radical scavenging (colorimetric)	Kiazist TAC kit (Kiazist Life Sciences, Tehran, Iran)	0–5 mmol/L	4.3
**Inflammation**	IL-6	High-sensitivity ELISA	Pishtaz Teb Zaman ELISA kit (Pishtaz Teb Zaman Diagnostics, Tehran, Iran)	0.16–10 pg/mL	6.2
	IL-10	High-sensitivity ELISA	Pishtaz Teb Zaman ELISA kit (Pishtaz Teb Zaman Diagnostics, Tehran, Iran)	0.5–50 pg/mL	5.8
	TNF-α	High-sensitivity ELISA	Pishtaz Teb Zaman ELISA kit (Pishtaz Teb Zaman Diagnostics, Tehran, Iran)	0.5–32 pg/mL	5.5
**Hormones**	Cortisol	Competitive ELISA	Monobind Inc. kit (distributed by Tosee Sabz Tamim, Tehran, Iran)	0.5–60 μg/dL	4.5
	Testosterone	Competitive ELISA	Monobind Inc. kit (distributed by Tosee Sabz Tamim, Tehran, Iran)	0.1–16 ng/mL	5.0
**Immune subsets**	CD4	Flow cytometry (T-helper lymphocytes)	BD FACSCanto II (Becton Dickinson, Franklin Lakes, NJ, USA) with Pishtaz Teb Zaman antibodies	‒	3.8
	CD8	Flow cytometry (cytotoxic T-lymphocytes)	BD FACSCanto II (Becton Dickinson, Franklin Lakes, NJ, USA) with Pishtaz Teb Zaman antibodies	‒	4.0
**Immunoglobulins**	IgA	Immunonephelometry	Siemens BN ProSpec (Siemens, Erlangen, Germany)	25–500 mg/dL	3.1
	IgG	Immunonephelometry	Siemens BN ProSpec (Siemens, Erlangen, Germany)	200–1800 mg/dL	2.8

Notes: CV = coefficient of variation; IFCC = International Federation of Clinical Chemistry; TBARS = thiobarbituric acid reactive substances; ABTS = 2,2′-azino-bis (3-ethylbenzothiazoline-6-sulfonic acid); ELISA = enzyme-linked immunosorbent assay.

**Table 2:** Baseline demographic and physical characteristics (mean ± SD).

Variable	Placebo (n = 8)	CoQ10 (n = 8)	Taurine (n = 8)	*p*-Value
**Age (years)**	17.9 ± 0.8	18.0 ± 0.8	17.8 ± 0.7	0.82
**Height (cm)**	175.9 ± 3.2	176.1 ± 3.0	175.3 ± 2.8	0.91
**Weight (kg)**	68.1 ± 3.0	68.6 ± 2.9	67.9 ± 2.8	0.86
Predicted VO_2_max (mL/kg/min) *	52.3 ± 1.9	53.1 ± 1.8	52.5 ± 1.7	0.63

* Estimated using the Queen’s College Step Test formula [26,27].

**Table 3:** Dietary intake analysis (mean ± SD) and between-group comparisons at baseline and post-intervention.

Nutrient	Time Point	Placebo (n = 8)	CoQ10 (n = 8)	Taurine (n = 8)	Group *p*-Value	Time *p*-Value	Group × Time *p*-Value
**Total energy** **intake (kcal)**	Baseline	2735 ± 310	2780 ± 325	2745 ± 295	0.82	0.67	0.91
	Post-intervention	2710 ± 345	2755 ± 335	2760 ± 310			
**CHO (g·kg^−1^·d^−1^)**	Baseline	4.9 ± 0.6	5.0 ± 0.5	4.8 ± 0.5	0.75	0.89	0.78
	Post-intervention	4.8 ± 0.7	4.9 ± 0.6	5.0 ± 0.5			
**PRO (g·kg^−1^·d^−1^)**	Baseline	1.6 ± 0.2	1.6 ± 0.2	1.7 ± 0.2	0.68	0.55	0.83
	Post-intervention	1.6 ± 0.2	1.7 ± 0.2	1.6 ± 0.1			
**Total lipids (g·kg^−1^·d^−1^)**	Baseline	1.4 ± 0.2	1.4 ± 0.3	1.4 ± 0.2	0.89	0.71	0.88
	Post-intervention	1.4 ± 0.2	1.3 ± 0.2	1.4 ± 0.2			
**SFA (g)**	Baseline	27.4 ± 6.2	28.1 ± 5.9	27.9 ± 6.5	0.92	0.80	0.94
	Post-intervention	26.9 ± 5.8	27.5 ± 6.1	27.2 ± 6.0			
**Cholesterol (mg)**	Baseline	342 ± 68	355 ± 72	348 ± 65	0.79	0.62	0.87
	Post-intervention	335 ± 70	348 ± 68	340 ± 70			

Notes: CHO = carbohydrates; PRO = protein; SFA = saturated fatty acids. No significant differences (*p* > 0.05) were found between groups at baseline, between groups at post-intervention, or within groups across the study period. Dietary data were collected using two-day food records and analysed with the SAMAR nutritional analysis software, which has been validated for the Iranian food database [31].

**Table 4:** Percent change in plasma volume (Dill–Costill)—mean ± SD.

Time	Placebo	CoQ10	Taurine	*p* (Group × Time)
**T1**	–13.2 ± 1.5	–7.0 ± 1.1 *†	–9.2 ± 1.3 *	<0.001
**T2**	–15.1 ± 1.8	–8.5 ± 1.2 *†	–11.0 ± 1.4 *	<0.001
**T3**	–2.5 ± 1.0	–1.0 ± 0.8	–1.8 ± 0.9	0.08

* *p* < 0.05 vs. placebo; † *p* < 0.05 vs. taurine.

**Table 5:** MDA (μmol/L) and TAC (mmol/L)—mean ± SD.

Marker	Group	T0	T1	T2	T3
**MDA**	Placebo	0.52 ± 0.02	0.99 ± 0.03 †	1.24 ± 0.03 †‡	0.69 ± 0.02 *
	CoQ10	0.52 ± 0.02	0.71 ± 0.02 *†	0.83 ± 0.02 *†‡	0.59 ± 0.02 *
	Taurine	0.51 ± 0.02	0.76 ± 0.02 *†	0.93 ± 0.03 *†‡	0.60 ± 0.02 *
**TAC**	Placebo	1.26 ± 0.02	1.05 ± 0.02 †	0.95 ± 0.02 †‡	1.18 ± 0.02 *
	CoQ10	1.26 ± 0.02	1.18 ± 0.02 *	1.14 ± 0.02 *	1.24 ± 0.02
	Taurine	1.27 ± 0.02	1.15 ± 0.02 *†	1.08 ± 0.02 *†	1.22 ± 0.02

† *p* < 0.05 vs. T0 within group; ‡ *p* < 0.05 vs. T1; * *p* < 0.05 vs. placebo at the same time point.

**Table 6:** CK (U/L) and LDH (U/L)—mean ± SD.

Marker	Group	T0	T1	T2	T3
**CK**	Placebo	121 ± 6	344 ± 11 †	514 ± 14 †‡	283 ± 12 *
	CoQ10	121 ± 6	214 ± 8 *†	284 ± 9 *†‡	163 ± 6 *
	Taurine	119 ± 6	228 ± 9 *†	315 ± 10 *†‡	168 ± 7 *
**LDH**	Placebo	281 ± 8	522 ± 12 †	684 ± 12 †‡	357 ± 10 *
	CoQ10	280 ± 7	384 ± 9 *†	434 ± 9 *†‡	303 ± 7 *
	Taurine	282 ± 8	399 ± 10 *†	468 ± 11 *†‡	312 ± 8 *

† *p* < 0.05 vs. T0; ‡ *p* < 0.05 vs. T1; * *p* < 0.05 vs. placebo at the same time point. At T2, CoQ10 reduced LDH by 37% vs. placebo and by 7% vs. taurine (*p* = 0.03).

**Table 7:** IL-6, IL-10, TNF-α (pg/mL)—mean ± SD.

Marker	Group	T0	T1	T2	T3
**IL-6**	Placebo	1.8 ± 0.1	5.3 ± 0.3 †	7.6 ± 0.3 †‡	3.3 ± 0.2 *
	CoQ10	1.8 ± 0.1	2.7 ± 0.2 *†	3.5 ± 0.2 *†‡	2.1 ± 0.1 *
	Taurine	1.8 ± 0.1	2.7 ± 0.2 *†	3.9 ± 0.2 *†‡	2.2 ± 0.1 *
**IL-10**	Placebo	4.2 ± 0.1	5.8 ± 0.3 †	6.6 ± 0.3 †‡	5.3 ± 0.2 *
	CoQ10	4.1 ± 0.1	5.0 ± 0.2 *†	5.7 ± 0.2 *†‡	4.6 ± 0.2
	Taurine	4.1 ± 0.1	6.2 ± 0.2 *†	7.5 ± 0.2 *†‡	5.7 ± 0.2 *
**TNF-α**	Placebo	2.1 ± 0.1	3.9 ± 0.2 †	4.9 ± 0.2 †‡	3.0 ± 0.2 *
	CoQ10	2.1 ± 0.1	2.9 ± 0.2 *†	3.3 ± 0.2 *†‡	2.4 ± 0.1 *
	Taurine	2.0 ± 0.1	3.0 ± 0.2 *†	3.6 ± 0.2 *†‡	2.5 ± 0.1 *

† *p* < 0.05 vs. T0; ‡ *p* < 0.05 vs. T1; * *p* < 0.05 vs. placebo at the same time point. At T2, taurine increased IL-10 by 14% vs. placebo (*p* = 0.01) and by 32% vs. CoQ10 (*p* = 0.005).

**Table 8:** Cortisol (μg/dL), testosterone (ng/dL), and T:C ratio (×10^−3^)—mean ± SD.

Marker	Group	T0	T1	T2	T3
**Cortisol**	Placebo	14.0 ± 0.3	26.7 ± 0.8 †	30.4 ± 0.8 †‡	18.7 ± 0.5 *
	CoQ10	14.0 ± 0.2	18.6 ± 0.5 *†	20.2 ± 0.6 *†‡	15.1 ± 0.4 *
	Taurine	14.0 ± 0.3	19.7 ± 0.6 *†	22.7 ± 0.7 *†‡	15.9 ± 0.5 *
**Testosterone**	Placebo	525 ± 8	472 ± 10 †	412 ± 12 †‡	480 ± 10 *
	CoQ10	524 ± 7	511 ± 8 *	494 ± 9 *	520 ± 7
	Taurine	525 ± 9	510 ± 9 *	495 ± 10 *	519 ± 8
**T:C ratio**	Placebo	37.5 ± 1.2	17.7 ± 1.2 †	13.6 ± 1.0 †‡	25.7 ± 1.3 *
	CoQ10	37.4 ± 1.1	27.5 ± 1.3 *†	24.5 ± 1.1 *†‡	34.5 ± 1.2 *
	Taurine	37.5 ± 1.2	25.9 ± 1.2 *†	21.8 ± 1.2 *†‡	32.7 ± 1.3 *

† *p* < 0.05 vs. T0; ‡ *p* < 0.05 vs. T1; * *p* < 0.05 vs. placebo at the same time point. At T2, CoQ10 maintained the T:C ratio 80% higher than placebo and 12% higher than taurine (*p* < 0.01 for both).

**Table 9:** CD4 count (cells/μL), CD8 count (cells/μL), and CD4/CD8 ratio—mean ± SD.

Marker	Group	T0	T1	T2	T3
**CD4**	Placebo	851 ± 9	723 ± 12 †	680 ± 15 †‡	800 ± 12 *
	CoQ10	849 ± 9	811 ± 11 *	790 ± 13 *	838 ± 10
	Taurine	850 ± 11	808 ± 12 *	795 ± 14 *	835 ± 12
**CD8**	Placebo	450 ± 8	385 ± 10 †	350 ± 12 †‡	415 ± 9 *
	CoQ10	452 ± 7	430 ± 9 *	416 ± 10 *	445 ± 8
	Taurine	451 ± 8	428 ± 10 *	412 ± 11 *	448 ± 9
**CD4/CD8**	Placebo	1.89 ± 0.07	1.88 ± 0.08	1.94 ± 0.10 *	1.93 ± 0.10 *
	CoQ10	1.88 ± 0.06	1.89 ± 0.07	1.90 ± 0.09	1.88 ± 0.07
	Taurine	1.89 ± 0.07	1.89 ± 0.08	1.93 ± 0.09	1.86 ± 0.08

† *p* < 0.05 vs. T0 within group; ‡ *p* < 0.05 vs. T1; * *p* < 0.05 vs. placebo at the same time point.

**Table 10:** IgA (mg/dL) and IgG (mg/dL)—mean ± SD.

Marker	Group	T0	T1	T2	T3
**IgA**	Placebo	128 ± 4	112 ± 5 †	98 ± 5 †‡	118 ± 4 *
	CoQ10	127 ± 3	122 ± 4	118 ± 4 *	125 ± 3
	Taurine	128 ± 4	121 ± 5	115 ± 5 *	126 ± 4
**IgG**	Placebo	1155 ± 12	985 ± 15 †	925 ± 18 †‡	1080 ± 14 *
	CoQ10	1154 ± 10	1102 ± 12 *	1080 ± 14 *	1140 ± 11
	Taurine	1156 ± 11	1090 ± 13 *	1065 ± 15 *	1128 ± 12

† *p* < 0.05 vs. T0; ‡ *p* < 0.05 vs. T1; * *p* < 0.05 vs. placebo at the same time point.

**Table 11:** Selected haematological variables at T2 (peak cumulative stress)—mean ± SD.

Variable	Placebo	CoQ10	Taurine	*p* (Group)
**Hct (%)**	48.2 ± 0.3	46.2 ± 0.2 *	47.3 ± 0.3 *†	<0.001
**Hgb (g/dL)**	16.2 ± 0.2	15.6 ± 0.1 *	15.9 ± 0.2 *†	<0.01
**Neutrophils (%)**	78 ± 2	68 ± 2 *	71 ± 2 *†	<0.001
**Lymphocytes (%)**	13 ± 1	22 ± 1 *	19 ± 1 *†	<0.001
**NLR**	6.0 ± 0.6	3.1 ± 0.2 *	3.9 ± 0.3 *†	<0.001
**Monocytes (%)**	5.5 ± 0.5	6.0 ± 0.4	5.5 ± 0.5	0.12
**Eosinophils (%)**	1.2 ± 0.3	1.8 ± 0.2 *	1.5 ± 0.2	<0.05

* *p* < 0.05 vs. placebo; † *p* < 0.05 CoQ10 vs. taurine. NLR = neutrophil-to-lymphocyte ratio.

## Data Availability

The datasets generated and analysed during this study are available from the corresponding author upon reasonable request. The data are not publicly available due to privacy or ethical restrictions related to the participant consent obtained for this study.

## Supplementary Materials

The following supporting information can be downloaded at: https://www.mdpi.com/article/10.3390/nu18142229/s1, Figure S1: CONSORT flow diagram of participant recruitment, randomisation, and analysis.
