# Supplementary figures and images for: Practical Nutritional Strategies to Attenuate Physiological Stress in Adolescent Soccer Players: A Comparative Trial of CoQ10 and Taurine
Source: Nutrients. 2026 Jul 9;18(14):2229. doi: 10.3390/nu18142229 (PMC13415384; doi:10.3390/nu18142229)

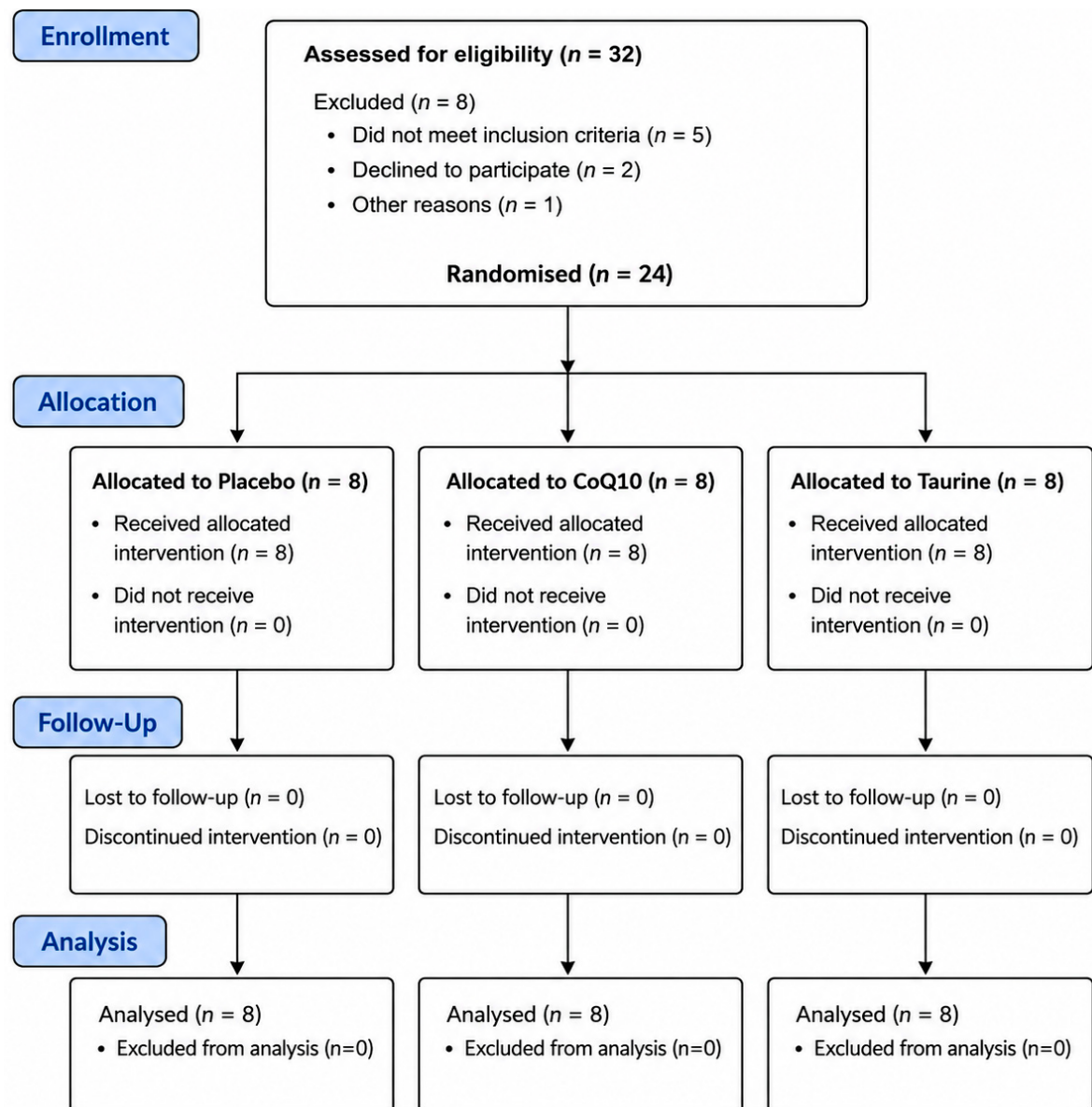

**Figure S1.** CONSORT flow diagram of participant recruitment, randomisation, and analysis.

Supplement: Supplementary file 1 [file nutrients-18-02229-s001.zip › nutrients-4379114-supplementary.pdf]
